# Chronic Cognitive Deficits and Associated Histopathology Following Closed-Head Concussive Injury in Rats

**DOI:** 10.3389/fneur.2019.00699

**Published:** 2019-07-02

**Authors:** Ying Deng-Bryant, Lai Yee Leung, Sindhu Madathil, Jesse Flerlage, Fangzhou Yang, Weihong Yang, Janice Gilsdorf, Deborah Shear

**Affiliations:** ^1^Brain Trauma Neuroprotection and Neurorestoration Branch, Center for Military Psychiatry and Neuroscience, Walter Reed Army Institute of Research, Silver Spring, MD, United States; ^2^Department of Surgery, Uniformed Services University of the Health Sciences, Bethesda, MD, United States

**Keywords:** traumatic brain injury, concussion, cognition, neurobehavior, neurodegeneration

## Abstract

Close-head concussive injury, as one of the most common forms of traumatic brain injury (TBI), has been shown to induce cognitive deficits that are long lasting. A concussive impact model was previously established in our lab that produces clinically relevant signs of concussion and induced acute pathological changes in rats. To evaluate the long-term effects of repeated concussions in this model, we utilized a comprehensive Morris water maze (MWM) paradigm for cognitive assessments at 1 and 6 months following repeated concussive impacts in rats. As such, adult Sprague-Dawley rats received either anesthesia (sham) or repeated concussive impacts (4 consecutive impacts at 1 h interval). At 1 month post-injury, results of the spatial learning task showed that the average latencies to locate the hidden “escape” platform were significantly longer in the injured rats over the last 2 days of the MWM testing compared to sham controls (*p* < 0.05). In the memory retention task, rats subjected to repeated concussive impacts also spent significantly less time in the platform zone searching for the missing platform during the probe trial (*p* < 0.05). On the working memory task, the injured rats showed a trend toward worse performance, but this failed to reach statistical significance compared to sham controls (*p* = 0.07). At 6 months post-injury, no differences were detected between the injured group and sham controls in either the spatial learning or probe trials. However, rats with repeated concussive impacts exhibited significantly worsened working memory performance compared to sham controls (*p* < 0.05). In addition, histopathological assessments for axonal neurodegeneration using silver stain showed that repeated concussive impacts induced significantly more axonal degeneration in the corpus callosum compared to sham controls (*p* < 0.05) at 1 month post-injury, whereas such difference was not observed at 6 months post-injury. Overall, the results show that repeated concussive impacts in our model produced significant cognitive deficits in both spatial learning abilities and in working memory abilities in a time-dependent fashion that may be indicative of progressive pathology and warrant further investigation.

## Introduction

Mild traumatic brain injury (mild TBI) or concussion has been reported to occur to an estimated 42 million people worldwide annually (1). While many patients showed improvements and returned to work within days or months after the injury, reportedly 22–36% suffered from prolonged cognitive impairments that lasted months and years beyond the initial injury (2). As such, there have been empirical data suggesting that specialized treatment is needed to improve the long-term outcomes of mild TBI patients (3). However, it remains challenging to delineate contributing factors that underlie various post-concussion symptoms and explore targeted treatments (4).

To better understand the neurobiology of concussion, we have previously developed and refined a closed-head concussive rat model that simulates a projectile impact concussion in humans and produces concussion-like clinical symptoms, such as loss of consciousness (LOC) (5). Compared to other existing mild TBI animal models (6–8), the WRAIR projectile concussive impact (PCI) model does not require scalp incision or craniotomy, which more closely resembles the real scenario of a close-head concussion. In addition, the PCI device produces a projectile impact at the rat head and the rat's head and cervical spine are allowed to move freely upon the impact, which commonly occurs in a true concussive event. Biomechanical calibration on model parameters, such as projectile mass, impact energy, and head movement kinetics, indicates that this model produces highly consistent and reproducible close-head concussive impacts in rats, making it an optimal exploratory platform for studying concussion preclinically (5).

Using the PCI model, we have previously conducted preliminary neurobiological assessments following a single vs. repeated concussive impacts for up to four impacts at 1 h intervals (5). The duration of loss of righting reflex, which indicates LOC, increases as the number of repeated impacts increases, suggesting increases in injury severity. It is noted that four consecutive concussive impacts produced more severe symptoms than a single impact did but remained to be a mild TBI, indicated by the duration of loss of righting reflex at <15 min (9). Anatomical examination of the brain tissues after four consecutive impacts in the PCI model showed that they were free of gross pathology, also indicating a mild TBI. Given concussive events, such as sports concussions, could occur repeatedly within a short time frame, a repetitive concussive model that results in a mild TBI would have important clinical relevance and more likely to lead to long lasting, rather than transient, cognitive deficits. Therefore, we examined the chronic cognitive deficits in rats subjected to repeated concussive impacts in the PCI model using Morris water maze (MWM).

The MWM is commonly used in animal models for assessing neurobehavior, especially spatial learning ability and memory function (10). It has a wide range of applications in understanding cognitive dysfunctions in neurodegenerative diseases, such as aging and neurotrauma. Different testing paradigms in the MWM have been designed to evaluate various aspects of cognitive dysfunctions in animal models of TBI. For example, retrograde memory loss was detected using MWM memory retention tests in rats that received a lateral fluid percussion injury (FPI), and such memory dysfunction was correlated to ipsilateral hippocampal cell loss (11). When the impact location was changed to the sagittal suture to generate a central FPI, rats performed worse than the controls in the MWM working memory test (12). In another study, the long-term MWM spatial learning disability in rats that sustained contusive impacts to the medial prefrontal cortex was suggested to be the result of attentional deficits due to TBI (13). It has also been reported that rats' inability to initiate search strategies in the MWM spatial learning task was linked to prefrontal cortical lesions as a result of penetrating injuries to the brain (14). More recently, methods in cognitive evaluation using MWM in neurotrauma have been summarized based on data produced in our lab (15). In the current study, we have re-designed some of the testing parameters for a more sensitive measurement of the spatial acquisition ability, memory retention, and working memory at chronic time points following repeated concussive impacts. Additionally, histological assessment for axonal neurodegeneration was conducted in rats that have completed the behavioral testing to explore possible pathological mechanisms that underlie long-term behavioral changes.

## Methods

### Subjects

Male adult Sprague-Dawley rats (48 rats total; 280–320 g; Charles River Labs, Raleigh, NC, USA) were used in these experiments. All procedures involving animal use were reviewed and approved by the Institutional Animal Care and Use Committee (IACUC) of Walter Reed Army Institute of Research. Research was conducted in compliance with the Animal Welfare Act and other federal statutes and regulations relating to animals and experiments involving animals and adheres to principles stated in the Guide for the Care and Use of Laboratory Animals, NRC Publication, 2011 edition. Animals were housed individually under a 12 h light/dark cycle in a facility accredited by the Association for Assessment and Accreditation of Laboratory Animal Care International (AAALACI).

### PCI Model

The PCI-induced concussive injury was described previously (5). The PCI apparatus consists of an elevated platform and a computer-controlled electro-pneumatic pressure release system used to launch a small projectile (i.e., a 3.52 g stainless steel sphere) targeted at the rat's head. Following anesthetization with 4% isoflurane, a custom-designed helmet (Army Research Lab, Aberdeen Proving Ground, MD) was securely fastened onto the rat's head. The anesthetized rat was placed on the elevated platform with its head positioned above an oval opening in the elevated platform such that the helmet-protected head was exposed to the projectile. A computer program was used to trigger the targeted release of the projectile at an operating pressure of 80 psi and produce a concussion targeting the right frontal region of the rat brain. Immediately following the concussive impact, the helmet was removed, and the rat was returned to its home cage. Rats were subjected to 4 repeated concussive impacts, spaced at 1 h apart, to produce more severe concussive symptoms, yet remaining within the limits of the mild TBI spectrum (5). Prior to each impact, rats were anesthetized, and the time of anesthesia was kept constant. The sham control rats received the same procedures (4 times anesthesia and air puffs, 1 h apart) except the projectile impact.

### Morris Water Maze (MWM) Task

**Figure 1:**
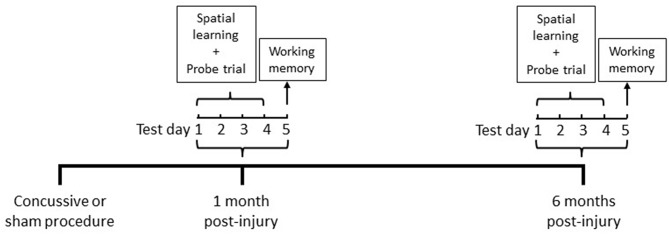
Timeline of MWM chronic cognitive testing paradigm.

Cognitive abilities were assessed in the MWM (Noldus EthoVision XT, VA) at 1 and 6 months in the same animals following repeated concussive impacts or sham procedures (Figure 1).

The MWM apparatus consists of a circular basin (75 cm deep; 175 cm diameter) filled with clear water (22°C room temperature) to a depth of 60 cm placed in a dark room with visual cues. A clear, plexiglas platform was submerged to a depth of 2.5 cm from the water surface and placed in the center of the northwest quadrant of the pool. The platform position remained constant during the spatial acquisition testing paradigm. Rats were placed in the pool at one of the equally spaced starting positions (north, south, east, and west). The starting position was pseudo-randomly determined for each trial within a day, alternating between short- and long-arms in reference to the platform. Each rat was allowed to swim freely to find the hidden platform or until 60 s elapsed. Rats were given four trials per day (5 min inter-trial interval) for 4 consecutive days. Mean latency to find the hidden platform were recorded on each day. A probe trial for testing memory retention was given on the last day immediately following the last trial of the spatial acquisition test. Each rat was allowed to swim freely until 60 s elapsed. Percent time spent in the platform zone searching for the missing platform during the probe trial was recorded. On testing day five, rats were given two sets of “one-trial learning” working memory test. Each set of the trials consist of two trials with 4 min inter-trial interval, in which the rats were given two opportunities to swim freely to search for the hidden platform or until 60 s elapsed. Platform location and starting positions for each trial set were determined pseudo-randomly. Latency difference (delta) to locate the platform between two trials within a trial set during the working memory test was recorded.

### Histology

At 1 month following repeated concussive impacts or sham procedures, a subgroup of animals (*n* = 12/time point/group) were euthanized after completing the MWM tasks, while the remaining animals were euthanized after they were tested again in the MWM at 6 months post-injury (*n* = 12/time point/group). For histological studies, rats were transcardially perfused with phosphate-buffered saline (PBS), followed by 4% cold paraformaldehyde under deep anesthesia. Coronal brain sections (40 μm) were cut from +3.72 to −6.84 mm anteroposterior from Bregma. Four sets of serial sections were collected at 960-μm intervals. All the samples were processed at FD NeuroTechnologies (Ellicott City, MD). The first set was processed for the detection of neurodegeneration with FD NeuroSilver™ Kit II (FD Neurotechnologies, Ellicott City, MD) according to the manufacturer's instructions. The remaining brain sections were stored for other histopathology assessments. The sections were mounted on microscope slides and cover-slipped with Permount (Fisher Scientific, Fair Lawn, NJ). Investigators blinded to the injury conditions took images of the sections using an Olympus VS120 Whole Slide Scanning System (Olympus Corporation of the Americas, Waltham, MA) at uniform criteria for sensitivity and exposure time. Silver positively stained cells were quantified using threshold analysis in the corpus callosum to evaluate neurodegeneration. The threshold value was set to consistently detect maximal positive staining of silver. To ensure objective quantification, the same threshold value was applied to all brain sections. All quantification was performed by an investigator blinded to the groups using Image J (NIH, Version 1.49).

### Statistical Analysis

Statistical analysis was performed using SAS software v9.1 (SAS Institute, NC) and SigmaPlot 12.0 (Systat Software Inc., CA). Two-way repeated measures analysis of variance (ANOVA) was used to analyze the behavioral data. One-way ANOVA was used to compare the data obtained from the probe trial of Morris water maze. Student's *t*-test was used to determine the between-group difference in histology data. The significance criterion of all the statistical tests was set at *p* < 0.05. Data are presented as the mean ± standard error of means (SEM).

## Results

### Spatial Acquisition Task

**Figure 2:**
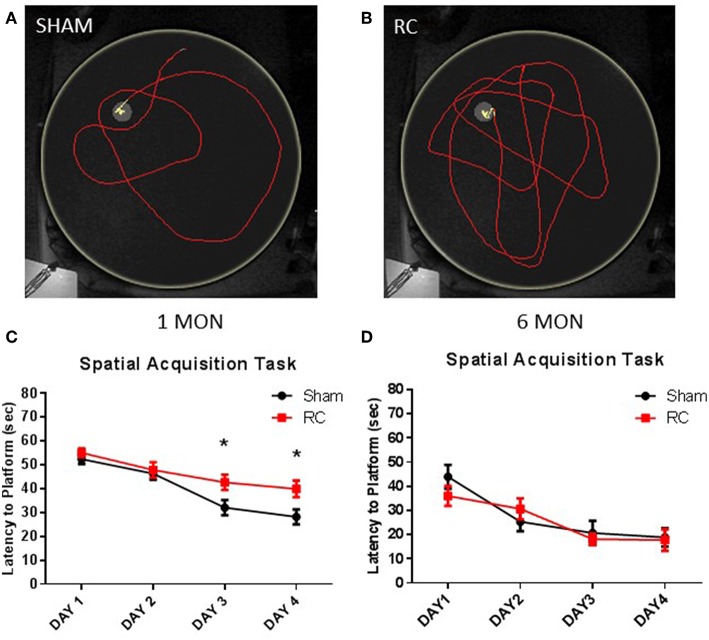
Spatial acquisition task. Trace within the MWM tank (yellow circle) represents swimming to search for the hidden platform to escape from the water (yellow trace—platform; red trace—outside of platform) for a sham **(A)** or a repeated concussed rat **(B)**. At 1 month post-injury **(C)**, results showed that the average latencies to locate the hidden “escape” platform were significantly longer in rats sustained repeated concussive impacts (RC) over the last 2 days of the MWM testing compared to sham controls (group *n* = 24; *p < 0.05). At 6 months post-injury **(D)**, no differences were detected between concussed rats (RC) and sham controls (group *n* = 12).

Spatial acquisition learning ability was assessed in the MWM at 1 and 6 months following sham procedure or repeated concussive impacts (Figure 2). In this task, the rats were allowed to swim freely to locate the platform and escape from the water using only spatial cues in a dark room. Representative trace images showed that a sham control rat (Figure 2A) quickly located the platform using visual cues, whereas rats that sustained repeated concussive impacts (Figure 2B) swam longer distance in searching for the hidden platform relatively aimlessly. Group average of mean latency (sec) to locate the platform per day was calculated. At 1 month post-injury, the sham controls showed better ability to navigate and spent significantly less amount of time to locate the platform on the 3rd and 4th day of the spatial acquisition task compared to the concussed rats (*p* < 0.05; two-way repeated ANOVA with S-N-K *post-hoc* test) (Figure 2C). At 6 months post-injury, the earlier observed superior searching ability in the sham control rats was lost and they exhibited similar spatial learning ability as the rats sustained concussive impacts (Figure 2D).

### Memory Retention Task

**Figure 3:**
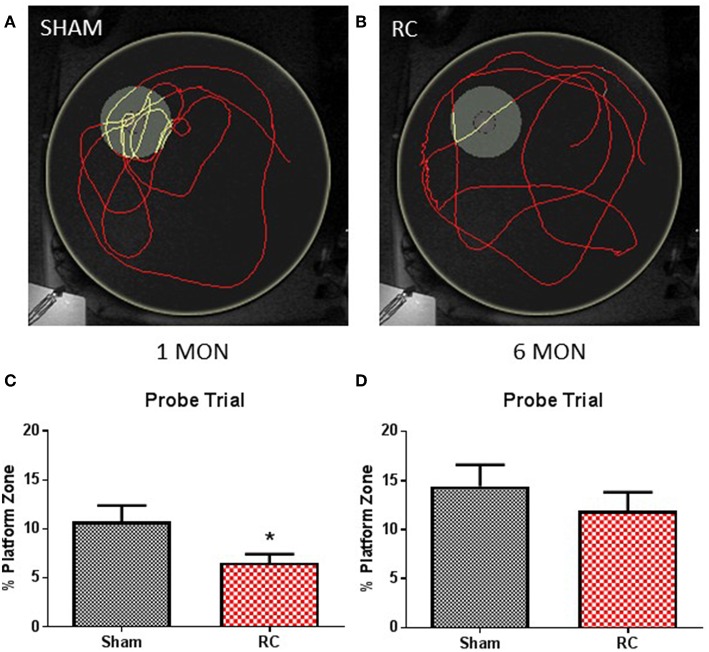
Memory retention task. Trace within the MWM tank (yellow circle) represents swimming to search for the missing platform during the probe trial (yellow trace—platform; red trace—outside of platform) for a sham **(A)** or a repeated concussed rat **(B)**. At 1 month post-injury **(C)**, rats sustained repeated concussive impacts (RC) spent significantly less time in the platform zone searching for the missing platform during the probe trial in the memory retention task (group *n* = 12; *p < 0.05). At 6 months post-injury **(D)**, no differences were detected between the concussed rats (RC) and sham controls (group *n* = 12).

Memory retention ability was evaluated during a probe trial in the MWM at 1 and 6 months following sham procedure or repeated concussive impacts (Figure 3).

At the end of the spatial acquisition assessment, the hidden platform was removed from the water tank, and rats were put back into the maze to search for the missing platform based on their recent memory. Representative trace images indicate that a sham control rat (Figure 3A) exhibited better memory retention and spent more time circling in the platform area to escape from the water, whereas a concussed rat (Figure 3B) distributed its search for the platform evenly throughout the water maze. Group average of percent (%) time out of 60 s that the rats spent in the platform zone was recorded and calculated. At 1 month post-injury, the concussed rats spent a significant less percentage of time in the platform zone compared to sham controls (*p* < 0.05; Student's *t*-test) (Figure 3C). At 6 months post-injury, both sham and rats subjected to repeated concussive impacts exhibited similar performance in the probe trial (Figure 3D).

### Working Memory Task

**Figure 4:**
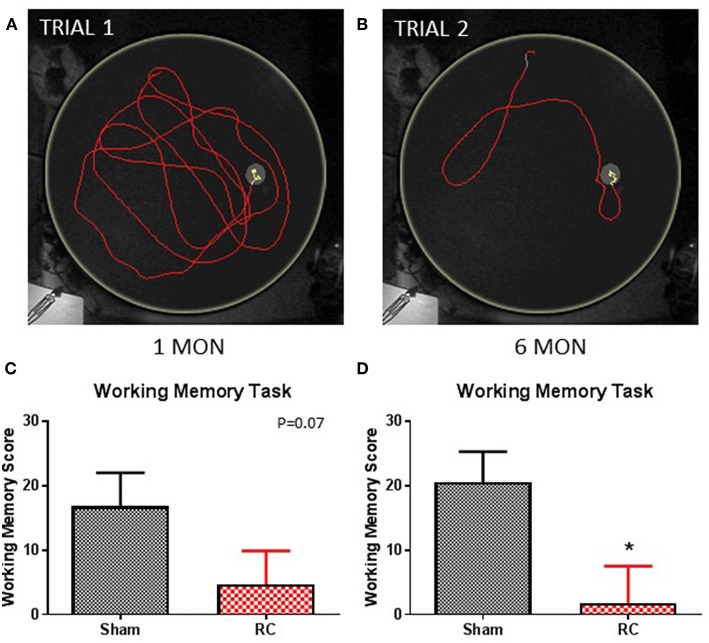
Working memory task. Trace within the MWM tank (yellow circle) represents swimming to search for the hidden platform in a set of trials: trial 1 **(A)** and trial 2 **(B)** (yellow trace—platform; red trace—outside of platform) for a sham rat. At 1 month post-injury **(C)**, rats sustained repeated concussive impacts (RC) showed a trend toward worse performance, but this failed to reach statistical significance compared to sham controls on the working memory task (group *n* = 12; *p* = 0.07). At 6 months post-injury **(D)**, the concussed rats (RC) exhibited significantly worsened working memory performance compared to sham controls (group *n* = 12; *p < 0.05).

Working memory function was assessed using a one-trial learning test in the MWM at 1 and 6 months following sham procedure or repeated concussive impacts (Figure 4).

In this test, paired trials (Trial 1 and Trial 2) with the same starting location and platform location was presented to the rats, in which the difference in time for the rats to locate the platform between the two trials was calculated. The latency different (Time_Trial1_-Time_Trial2_−) between two trials within a trial pair indicates the working memory ability, such that the larger the latency difference the better working memory function. The test was then repeated with a different pair of trials that have a different set of starting location and platform location from the previous trial pair. Representative trace images demonstrate that a rat spent more time in the first trial (Trial 1; Figure 4A) to locate the platform, while it spent less time when the trial (Trial 2; Figure 4B) was repeated immediately. Group average of the mean latency difference was calculated. At 1 month post-injury, concussed rats exhibited a trend toward worsened working memory function compared to sham controls (*p* = 0.07; Student's *t*-test) (Figure 4C). At 6 months post-injury, rats with repeated concussive impacts showed a significantly worse performance in the working memory task compared to sham controls (*p* < 0.05; Student's *t*-test) (Figure 4D).

### Histology

**Figure 5:**
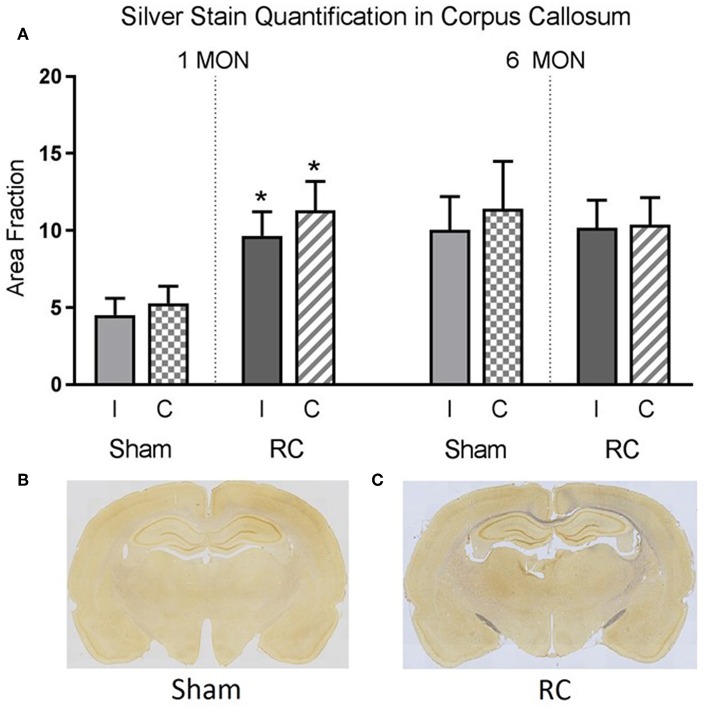
Quantification of neurodegeneration by silver staining in the corpus callosum. At 1 month post-injury, rats sustained repeated concussive impacts (RC) showed significantly increased levels of silver staining in both ipsilateral (I) and contralateral (C) corpus callosum compared to sham controls (group *n* = 12; *p < 0.05) **(A)**. At 6 months post-injury, concussed rats (RC) and sham controls showed the same level of silver staining in the corpus callosum **(A)**. Representative coronal brain sections showed the intensity of silver stain in sham **(B)** vs. concussed rats (RC) **(C)** at 1 month post-injury (group *n* = 12).

Silver staining for axonal neurodegeneration in the corpus callosum (Supplement Figure 1) was quantified at 1 and 6 months following sham procedure or repeated concussive impacts in rats (*n* = 12/time point/group) that completed the cognitive assessments (Figure 5).

Representative coronal brain sections showed an example of the intensity of the silver staining in sham (Figure 5B) vs. rats subjected to concussive impacts (Figure 5C). Area fraction of the silver staining in both ipsilateral and contralateral corpus callosum region was presented in the bar graph (Figure 5A). At 1 month post-injury, the results showed that silver staining was significantly increased in the concussed rats compare to the sham controls in both ipsilateral and contralateral corpus callosum (*p* < 0.05; Student's *t*-test). At 6 months post-injury, the silver staining levels in sham control rats elevated and showed no difference from that detected in the concussed rats.

## Discussion

The current study demonstrates that repeated concussive impacts produced by the PCI device resulted in long-term cognitive deficits in rats that are detectable using MWM. While the repeated impacts produced a mild TBI, the MWM paradigms designed in this study were shown to be sensitive for measuring small yet significant long-term cognitive differences between concussed rats and sham controls. In addition, rats sustained repeated concussive impacts demonstrated cognitive dysfunction in spatial learning at 1 month post-injury, while it progressed to working memory deficits at 6 months post-injury. Histopathological evaluation revealed that axonal degeneration in the corpus callosum may be related to cognitive dysfunction after repeated concussive impacts.

Cognitive deficits are often experienced by mild TBI patients during the acute phase following injuries and in many cases progress to long-term problems. Mild TBI patients have reported to experience numerous cognitive impairments, including having difficulty in learning and memory, and attention and information processing speed (16). In this study, results showed that rats that received repeated concussive impacts performed significantly worse compared to sham controls in the MWM spatial acquisition task and the probe trial at 1 month post-injury. In experimental models, spatial acquisition is thought to largely depend on hippocampal function for retaining spatial memory during the MWM trials (17). In support of that, the current study showed that the concussed rats' ability to retain memory during the probe trial at 1 month post-injury was also significantly impaired compared to sham controls. Additionally, it has been indicated that dorsal hippocampus is more susceptible to a blunt head impact and thus plays a more important role on spatial learning ability than the ventral side (18). However, the ability to acquire spatial learning strategy may not be limited to mnemonic function, but involves other aspects of the brain function, such as search strategies and attention. For example, lesions to medial thalamus in animal models revealed its important role in search strategies and swimming behavior (e.g., thigmotaxic swimming) in the MWM (19). In addition, frontal lobe injuries resulted in rats spending more time and swimming longer distance to find the hidden platform, indicating their inability to initiate search strategies in the MWM (20). Although repeated concussive impacts in our model produces a mild injury, it is possible that the high velocity projectile impact and the rapid rotation of the head led to impairment of brain functions that depend on frontal lobe and related circuitry. Consistent with that, clinical evidence has shown that frontal lobes and subcortical structures that support executive functions, such as planning, aspects of attention, and purposeful behavior, are vulnerable to injuries, leading to cognitive dysfunctions in mild TBI patients (21).

The MWM one-trial learning test showed that the concussed rats' performance trended worse compared to sham controls at 1 month and the effect reached statistical significance at 6 months post-injury. In this test, working memory function is required to store and process the trial-specific information for a short term in order to guide the navigation to the platform in the immediately repeated trial. Working memory process is thought to be largely involved with the prefrontal cortical region (22). As such, repeated impacts to the frontal lobe and related circuitry can also lead to working memory deficits, given that these areas appear to be susceptible to injuries in the PCI model as discussed above. Working memory function is essential for complex cognitive activities, including executive functions such as learning and reasoning (23). The cognitive processes that are used in the spatial acquisition task, such as planning, memory, and attention, involve some degree of working memory function. As such, working memory deficits may have contributed partially to the worse performance in the spatial acquisition task in rats that received repeated concussive impacts compared to sham controls. This explains the concurrent deficits in both spatial learning ability and working memory function observed at 1 month following repeated concussive impacts in the current study. Consistent with the observation in the rat PCI model, functional imaging data have shown that working memory capacity was impaired in mild TBI patients (24). It is noted that persistent working memory deficits can present without observable brain structural damage. Therefore, changes in neurotransmitters and receptors, along with the possible abnormality in cellular structures that develops over time, are suggested to be the underlying mechanisms for prolonged working memory dysfunction following TBI (25).

In the present study, MWM tasks were repeated in the same rats at 6 months post-injury in order to evaluate the progression of cognitive dysfunction following repeated concussive impacts. Notably, spatial learning and memory retention deficits observed in the concussed rats at 1 month post-injury was not detected at 6 months post-injury, while working memory dysfunction persisted through 6 months. This suggests cognitive recovery in the time spent in MWM to locate the hidden platform, while other aspects of cognitive deficits worsened. Similarly, Darwish et al. reported that rats showed normal latency to locate the platform in MWM, while impairments in search strategies and memory retention remained within 1 month following a mild TBI (26). Clinically, long-term effects of a mild TBI on cognitive performance are more complex. For example, cognitive dysfunction following sports-related concussion in some patients reverted to normalcy based on neurocognitive testing at around 2–3 months' time post-injury and yet self-reported symptoms persisted (27). While these self-reported chronic cognitive symptoms are not always reflected by the neurocognitive testing results, mild TBI patients with persistent symptoms showed significantly worse performance in working memory and information processing speed tasks than non-symptomatic mild TBI patients beyond 1 year after injury (28). Consistent with that, experimental TBI models also showed chronic deficits in working memory function that plays a key role in many high-level cognitive activities, including information processing speed and attention. However, working memory dysfunction can persist without overt brain structural damage during chronic phase following TBI. It is suggested that such dysfunction may be, in part, explained by the cellular and molecular mechanisms, such as the increased GABA-mediated inhibition of prefrontal neuronal activity (29). However, neurotransmission inhibition appeared to be acute and resolved in about 1 month post-injury. Alternatively, increased dendritic spine density and altered catecholamine signaling were purported to be responsible for working memory deficits during chronic phase following TBI (25). In this study, repeated concussive injury resulted in a time-dependent change in cognitive function that was detectable using different MWM tasks, each designed to evaluate specific cognitive functions including spatial learning ability and working memory function. As such, the PCI model may serve as an ideal platform for exploring mechanisms that underlie acute and chronic cognitive dysfunctions following close-head concussions.

Silver staining showed that neurodegeneration significantly increased in the corpus callosum in the concussed rats compared to sham controls at 1 month and this level of silver staining sustained out to 6 months following injury. There have been clinical evidence indicating white matter track abnormalities following sports-related concussions in retired athletes, which was purported to associate with cognitive deficits (30). In addition, pronounced atrophy was detected in the corpus callosum in TBI patients who survived the injury after 1 year (31). However, the link between white matter track abnormalities and chronic cognitive deficits remains unclear. It has been purported that axonal demyelination occurs in the corpus callosum, or there have been anterograde degeneration through corpus callosum from ipsilateral degenerating neurons. In addition, chronic inflammation was observed within the corpus callosum in mild TBI models (32, 33). Thus, one of the limitations of this study is that neuroinflammatory responses, such as microglial reactivity, were not investigated. Another limitation would be that cellular mechanisms underlying potential axonal demyelination of the commissural fibers within the corpus callosum were not examined. It should be noted that the silver staining in the corpus callosum in sham controls increased at 6 months compared to 1 month after sham procedure, reaching similar levels as that in rats subjected to repeated concussive impacts measured at 6 months after injury. Similarly, Onyszchuk et al. also reported increased neurodegeneration in the aged sham brains detected by silver staining (34). This study suggests that a more pronounced blood-brain barrier opening in the aged sham animals may have contributed to the increased neurodegeneration, which also positioned the aged brains more vulnerable to injuries (34). In the current study, an earlier onset (1 month) of increased neurodegeneration was detected in the concussed brains compare to the sham controls, but this did not progress when assessed at 6 months post-injury. Given that in this study all the animals received repeated concussive impacts or sham procedure were at the same age (i.e., adults), the mild injuries that the concussed rats sustained may have only expedited the onset of the neurodegeneration process that would have occurred later due to normal aging. However, further studies are warranted to confirm this hypothesis. For the increased axonal degeneration detected by silver staining in the corpus callosum area, there have been reports suggest that damage to callosal fibers can cause visual disturbances in animals (35, 36). Thus, the rats' MWM performance could be affected by potential impairments to the visual center of the brain, and not limited to memory deficits, indicating a possible limitation of this study. However, MWM data at 6 months post-injury showed that the sham controls and the concussed rats performed similarly in both spatial acquisition task and memory retention task, suggesting that any potential visual center damage may not have induced detectable differences in the MWM performance. In support of that, it has been reported that axonal damage in optic nerve and optic track areas did not induce noticeable effects on cognitive and motor outcomes (6). Nonetheless, given the higher level of silver staining in the corpus callosum, future studies are warranted to test visual center impairment following concussive injuries.

Previously, we have demonstrated that the PCI model produces clinically relevant concussion symptoms and acute pathological changes in rats. The current study showed that the PCI model also generates long lasting cognitive deficits that may reflect chronic cognitive impairments seen in mild TBI patients. More importantly, the PCI-induced cognitive deficits in spatial learning ability and working memory developed over time, which may indicate progressive changes in cellular and molecular mechanisms despite the lack of gross pathology. Further studies using this model would be informative with regard to investigating mechanistic factors that underlie chronic clinical symptoms following concussions.

## Ethics Statement

This study was carried out in accordance with the recommendations of Guide for the Care and Use of Laboratory Animals, NRC Publication, 2011 edition. The protocol was approved by the Institutional Animal Care and Use Committee (IACUC) of Walter Reed Army Institute of Research.

## Author Contributions

YD-B wrote the manuscript and designed the study. YD-B, LL, SM, JF, FY, WY, and DS contributed to data collection, data analysis, or interpretation. JG and DS reviewed the study design and data analysis, and edited the manuscript.

### Conflict of Interest Statement

The authors declare that the research was conducted in the absence of any commercial or financial relationships that could be construed as a potential conflict of interest.

## Supplementary Material

Supplement Figure 1 Higher magnification of silver staining in the corpus callosum. Silver staining indicates elevated levels of axonal degeneration in the corpus callosum region in rats received projectile concussive impact (PCI) than the sham controls at 6 months post-injury.

## References

[1] Gardner RC Yaffe K. Epidemiology of mild traumatic brain injury and neurodegenerative disease. Mol Cell Neurosci. (2015) 66(Pt B):75–80. 10.1016/j.mcn.2015.03.001 25748121 PMC4461453

[2] Donovan J Cancelliere C Cassidy JD. Summary of the findings of the International Collaboration on Mild Traumatic Brain Injury Prognosis. Chiropr Man Therap. (2014) 22:38. 10.1186/s12998-014-0038-3 25379171 PMC4221725

[3] Bertisch H Satris G Temkin N Barber J Manley GT Transforming R. Rehabilitation trajectories and outcomes in individuals with mild traumatic brain injury and psychiatric histories: a TRACK-TBI pilot study. J Head Trauma Rehabil. (2018) 34:36–44. 10.1097/HTR.0000000000000399 29863617

[4] Pineau H Marchand A Guay S. Specificity of cognitive and behavioral complaints in post-traumatic stress disorder and mild traumatic brain injury. Behav Sci. (2015) 5:43–58. 10.3390/bs5010043 25646994 PMC4384062

[5] Leung LY Larimore Z Holmes L Cartagena C Mountney A Deng-Bryant Y. The WRAIR projectile concussive impact model of mild traumatic brain injury: re-design, testing and preclinical validation. Ann Biomed Eng. (2014) 42:1618–30. 10.1007/s10439-014-1014-8 24756867

[6] Creeley CE Wozniak DF Bayly PV Olney JW Lewis LM. Multiple episodes of mild traumatic brain injury result in impaired cognitive performance in mice. Acad Emerg Med. (2004) 11:809–19. 10.1111/j.1553-2712.2004.tb00761.x 15289185

[7] Prins ML Hales A Reger M Giza CC Hovda DA. Repeat traumatic brain injury in the juvenile rat is associated with increased axonal injury and cognitive impairments. Dev Neurosci. (2010) 32:510–8. 10.1159/000316800 20829578 PMC3215244

[8] Shultz SR Bao F Omana V Chiu C Brown A Cain DP. Repeated mild lateral fluid percussion brain injury in the rat causes cumulative long-term behavioral impairments, neuroinflammation, and cortical loss in an animal model of repeated concussion. J Neurotrauma. (2012) 29:281–94. 10.1089/neu.2011.2123 21933013

[9] Dewitt DS Perez-Polo R Hulsebosch CE Dash PK Robertson CS. Challenges in the development of rodent models of mild traumatic brain injury. J Neurotrauma. (2013) 30:688–701. 10.1089/neu.2012.2349 23286417

[10] Morris R. Developments of a water-maze procedure for studying spatial learning in the rat. J Neurosci Methods. (1984) 11:47–60. 6471907 10.1016/0165-0270(84)90007-4

[11] Smith DH Okiyama K Thomas MJ Claussen B McIntosh TK. Evaluation of memory dysfunction following experimental brain injury using the Morris water maze. J Neurotrauma. (1991) 8:259–69. 10.1089/neu.1991.8.259 1803034

[12] Hamm RJ Temple MD Pike BR O'Dell DM Buck DL Lyeth BG. Working memory deficits following traumatic brain injury in the rat. J Neurotrauma. (1996) 13:317–23. 10.1089/neu.1996.13.317 8835799

[13] Lindner MD Plone MA Cain CK Frydel B Francis JM Emerich DF. Dissociable long-term cognitive deficits after frontal versus sensorimotor cortical contusions. J Neurotrauma. (1998) 15:199–216. 10.1089/neu.1998.15.199 9528920

[14] Shear DA Lu XC Bombard MC Pedersen R Chen Z Davis A. Longitudinal characterization of motor and cognitive deficits in a model of penetrating ballistic-like brain injury. J Neurotrauma. (2010) 27:1911–23. 10.1089/neu.2010.1399 20684676

[15] Deng-Bryant Y Leung LY Caudle K Tortella F Shear D. Cognitive evaluation using Morris water maze in neurotrauma. Methods Mol Biol. (2016) 1462:539–51. 10.1007/978-1-4939-3816-2_29 27604737

[16] Carroll LJ Cassidy JD Cancelliere C Cote P Hincapie CA Kristman VL. Systematic review of the prognosis after mild traumatic brain injury in adults: cognitive, psychiatric, and mortality outcomes: results of the International Collaboration on Mild Traumatic Brain Injury Prognosis. Arch Phys Med Rehabil. (2014) 95:S152–173. 10.1016/j.apmr.2013.08.300 24581903

[17] Morris RG Garrud P Rawlins JN O'Keefe J. Place navigation impaired in rats with hippocampal lesions. Nature. (1982) 297:681–3. 7088155 10.1038/297681a0

[18] Moser E Moser MB Andersen P. Spatial learning impairment parallels the magnitude of dorsal hippocampal lesions, but is hardly present following ventral lesions. J Neurosci. (1993) 13:3916–25. 8366351 10.1523/JNEUROSCI.13-09-03916.1993 PMC6576447

[19] Cain DP Boon F Corcoran ME. Thalamic and hippocampal mechanisms in spatial navigation: a dissociation between brain mechanisms for learning how versus learning where to navigate. Behav Brain Res. (2006) 170:241–56. 10.1016/j.bbr.2006.02.023 16569442

[20] Hoffman SW Fulop Z Stein DG. Bilateral frontal cortical contusion in rats: behavioral and anatomic consequences. J Neurotrauma. (1994) 11:417–31. 10.1089/neu.1994.11.417 7837282

[21] McDonald BC Flashman LA Saykin AJ. Executive dysfunction following traumatic brain injury: neural substrates and treatment strategies. NeuroRehabilitation. (2002) 17:333–44. 12547981

[22] Funahashi S. Prefrontal cortex and working memory processes. Neuroscience. (2006) 139:251–61. 10.1016/j.neuroscience.2005.07.003 16325345

[23] Baddeley A. Working memory: the interface between memory and cognition. J Cogn Neurosci. (1992) 4:281–8. 10.1162/jocn.1992.4.3.281 23964884

[24] McAllister TW Sparling MB Flashman LA Guerin SJ Mamourian AC Saykin AJ. Differential working memory load effects after mild traumatic brain injury. Neuroimage. (2001) 14:1004–12. 10.1006/nimg.2001.0899 11697932

[25] Hoskison MM Moore AN Hu B Orsi S Kobori N Dash PK. Persistent working memory dysfunction following traumatic brain injury: evidence for a time-dependent mechanism. Neuroscience. (2009) 159:483–91. 10.1016/j.neuroscience.2008.12.050 19167462 PMC4264540

[26] Darwish H Mahmood A Schallert T Chopp M Therrien B. Mild traumatic brain injury (MTBI) leads to spatial learning deficits. Brain Inj. (2012) 26:151–65. 10.3109/02699052.2011.635362 22360521 PMC3925503

[27] McCrea M Guskiewicz K Randolph C Barr WB Hammeke TA Marshall SW. Incidence, clinical course, and predictors of prolonged recovery time following sport-related concussion in high school and college athletes. J Int Neuropsychol Soc. (2013) 19:22–33. 10.1017/S1355617712000872 23058235

[28] Dean PJ Sterr A. Long-term effects of mild traumatic brain injury on cognitive performance. Front Hum Neurosci. (2013) 7:30. 10.3389/fnhum.2013.00030 23408228 PMC3569844

[29] Kobori N Dash PK. Reversal of brain injury-induced prefrontal glutamic acid decarboxylase expression and working memory deficits by D1 receptor antagonism. J Neurosci. (2006) 26:4236–46. 10.1523/JNEUROSCI.4687-05.2006 16624944 PMC6673989

[30] Multani N Goswami R Khodadadi M Ebraheem A Davis KD Tator CH The association between white-matter tract abnormalities, and neuropsychiatric and cognitive symptoms in retired professional football players with multiple concussions. J Neurol. (2016) 263:1332–41. 10.1007/s00415-016-8141-0 27142715

[31] Johnson VE Stewart JE Begbie FD Trojanowski JQ Smith DH Stewart W. Inflammation and white matter degeneration persist for years after a single traumatic brain injury. Brain. (2013) 136(Pt 1):28–42. 10.1093/brain/aws322 23365092 PMC3562078

[32] Shitaka Y Tran HT Bennett RE Sanchez L Levy MA Dikranian K. Repetitive closed-skull traumatic brain injury in mice causes persistent multifocal axonal injury and microglial reactivity. J Neuropathol Exp Neurol. (2011) 70:551–67. 10.1097/NEN.0b013e31821f891f 21666502 PMC3118973

[33] Gold EM Vasilevko V Hasselmann J Tiefenthaler C Hoa D Ranawaka K. Repeated mild closed head injuries induce long-term white matter pathology and neuronal loss that are correlated with behavioral deficits. ASN Neuro. (2018) 10:1759091418781921. 10.1177/1759091418781921 29932344 PMC6050992

[34] Onyszchuk G He YY Berman NE Brooks WM. Detrimental effects of aging on outcome from traumatic brain injury: a behavioral, magnetic resonance imaging, and histological study in mice. J Neurotrauma. (2008) 25:153–71. 10.1089/neu.2007.0430 18260798

[35] Yin TC Voorhees JR Genova RM Davis KC Madison AM Britt JK. Acute axonal degeneration drives development of cognitive, motor, and visual deficits after blast-mediated traumatic brain injury in mice. eNeuro. (2016) 3:ENEURO.0220-16.2016. 10.1523/ENEURO.0220-16.2016 27822499 PMC5086797

[36] Sen N. An insight into the vision impairment following traumatic brain injury. Neurochem Int. (2017) 111:103–7. 10.1016/j.neuint.2017.01.019 28163060 PMC5540824

