# Supplementary figures and images for: Chronic Cognitive Deficits and Associated Histopathology Following Closed-Head Concussive Injury in Rats
Source: Front Neurol. 2019 Jul 2;10:699. doi: 10.3389/fneur.2019.00699 (PMC6614177; doi:10.3389/fneur.2019.00699)

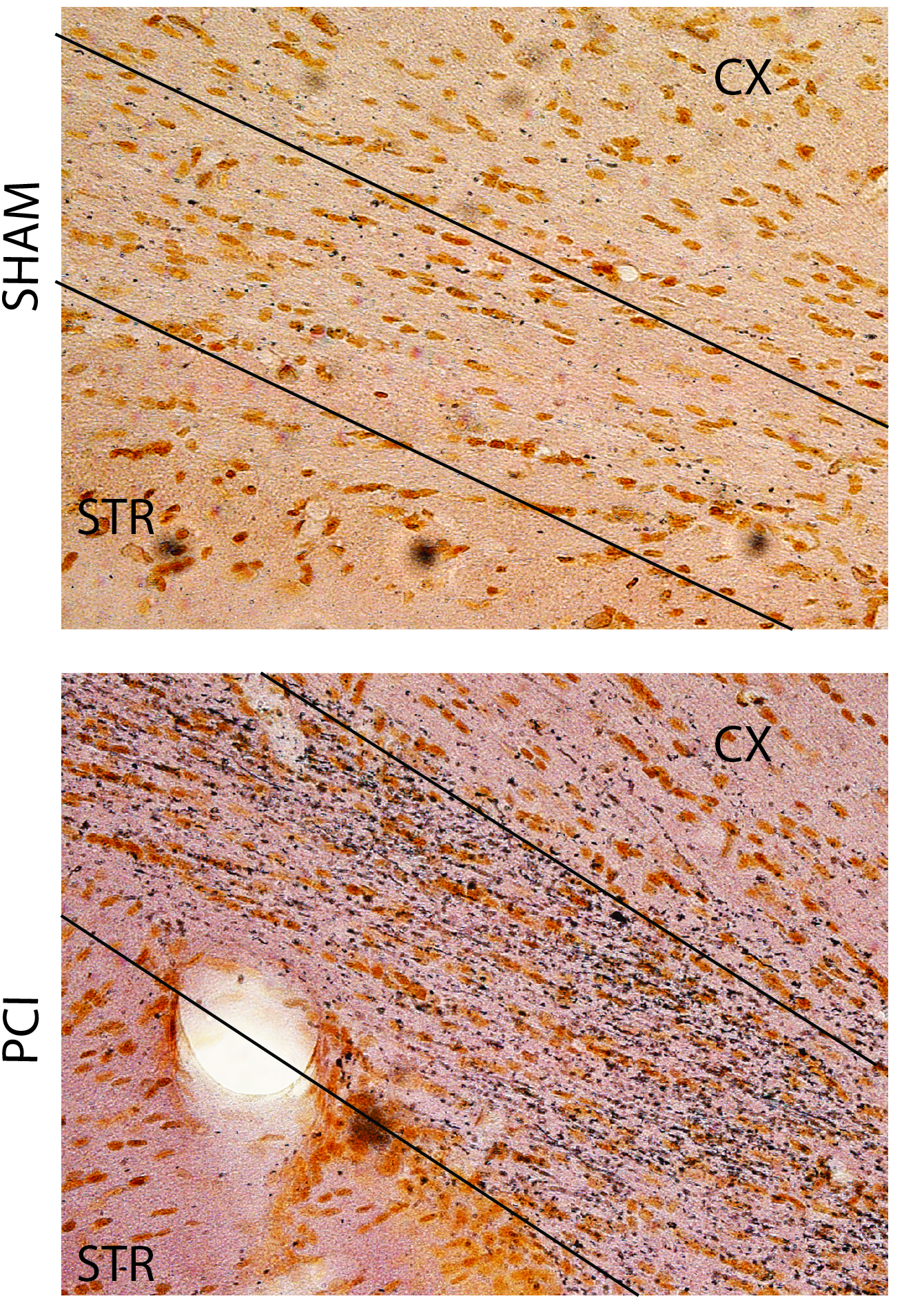

Supplement: Supplement Figure 1 — Higher magnification of silver staining in the corpus callosum. Silver staining indicates elevated levels of axonal degeneration in the corpus callosum region in rats received projectile concussive impact (PCI) than the sham controls at 6 months post-injury. [file Image_1.tif]
